# Simultaneous fecal microbial and metabolite profiling enables accurate classification of pediatric irritable bowel syndrome

**DOI:** 10.1186/s40168-015-0139-9

**Published:** 2015-12-09

**Authors:** Vijay Shankar, Nicholas V. Reo, Oleg Paliy

**Affiliations:** Department of Biochemistry and Molecular Biology, Boonshoft School of Medicine, Wright State University, Dayton, OH USA

**Keywords:** IBS, Children, Metabolomics, Microbiota, Diagnosis

## Abstract

**Background:**

We previously showed that stool samples of pre-adolescent and adolescent US children diagnosed with diarrhea-predominant IBS (IBS-D) had different compositions of microbiota and metabolites compared to healthy age-matched controls. Here we explored whether observed fecal microbiota and metabolite differences between these two adolescent populations can be used to discriminate between IBS and health.

**Findings:**

We constructed individual microbiota- and metabolite-based sample classification models based on the partial least squares multivariate analysis and then applied a Bayesian approach to integrate individual models into a single classifier. The resulting combined classification achieved 84 % accuracy of correct sample group assignment and 86 % prediction for IBS-D in cross-validation tests. The performance of the cumulative classification model was further validated by the de novo analysis of stool samples from a small independent IBS-D cohort.

**Conclusion:**

High-throughput microbial and metabolite profiling of subject stool samples can be used to facilitate IBS diagnosis.

**Electronic supplementary material:**

The online version of this article (doi:10.1186/s40168-015-0139-9) contains supplementary material, which is available to authorized users.

## Findings

Irritable bowel syndrome (IBS) is one of the most common disorders of the human gastrointestinal system affecting approximately 10-20 % of the population worldwide [1]. This syndrome affects young children, adolescents, and adults, with higher prevalence in adolescence [2]. IBS can be manifested by varied symptoms that include abdominal pain, changes in bowel habit, bloating and excessive flatus without visible damage to the intestinal mucosa, or high-level inflammation. Several different subtypes of IBS are recognized including diarrhea-predominant (IBS-D), constipation-predominant, mixed-type, and unsubtyped IBS [1, 3].

Proposed causes of IBS include increased intestinal permeability, food intolerance, altered motor function, abnormal gas handling, bacterial overgrowth of the small intestine, acute bacterial gastroenteritis, and altered immune response of the host [1, 3]. Several of these causes are linked to the functionality of human intestinal microbiota [4], and a number of recent studies have provided emerging evidence of gut microbiota alterations in IBS [5–8]. Gut microbes can affect the host directly through host-microbial interactions or indirectly through the transformation and production of organic compounds that are released into the intestinal lumen [9]. Thus, differences in microbial communities between healthy individuals and those with IBS can manifest themselves as disparities in luminal metabolite profiles, a hypothesis supported by several reports [10–12].

The variety of symptoms that can be associated with IBS and the lack of readily observable intestinal pathophysiology make the diagnosis of this syndrome challenging. Rome criteria serve as the current standard diagnostic tool in clinical trials [1, 3], while different types of questionnaires and lactulose or glucose hydrogen breath tests are sometimes used in the clinic to rule out alternative causes [3, 4, 13]. Because of symptom overlap with other disorders, IBS can often be misdiagnosed, and IBS patients undergo unnecessary invasive tests such as colonoscopy [14]. Therefore, finding additional criteria to define IBS would advance its diagnosis, lower medical costs, and improve patient outcomes [1].

**Fig. 1:**
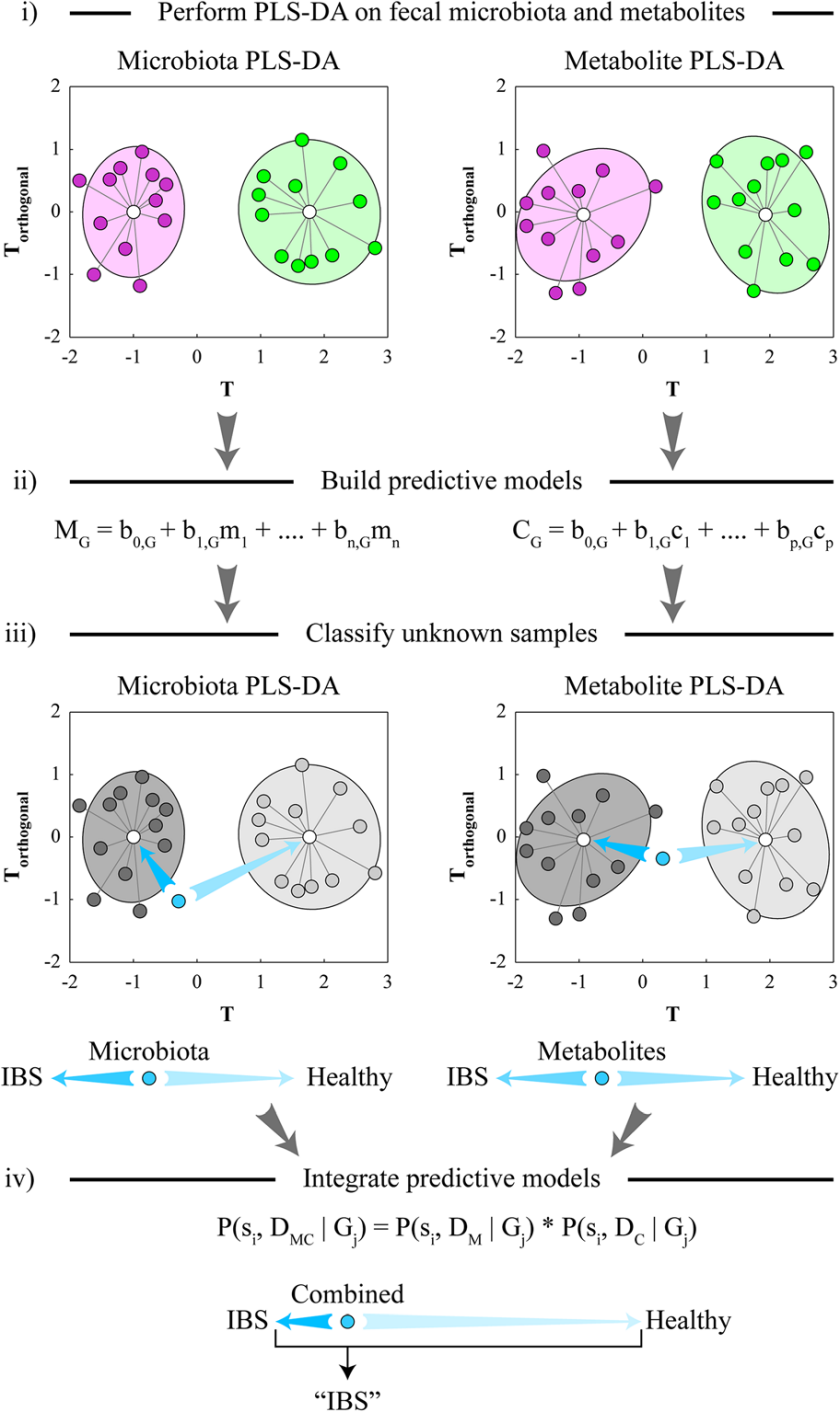
Schematic overview of the classification model generation. *Pink* and *green points* represent individual kIBS and kHLT samples, respectively, distributed in the simulated T-vs-T_orthogonal_ PLS ordination space. *Blue point* represents an unknown sample that is classified by the PLS-DA models. *M* and *C* denote overall microbiota- and metabolite-based classification models, respectively; *G* is the group identifier; *b*_*0…n*_ are numerical parameters and *m*_*1…n*_ and *c*_*1…p*_ are values of specific microbes and metabolites, respectively. See statistical data analyses section for the definitions of Bayesian model terms and parameters

In previous reports, we compared the fecal microbiota and metabolites of healthy pre-adolescent and adolescent children to those from children diagnosed with diarrhea-predominant IBS [6, 12, 15]. The fecal samples were obtained from 22 healthy children (average age = 12.6 years) and from 22 age-matched children with IBS-D (average age = 13.2 years) of both genders. Diagnosis of the IBS, inclusion and exclusion criteria, and stool collection procedure were described previously [6]. Phylogenetic Microbiota Array was used to obtain quantitative microbial phylotype and genus abundance values from all collected fecal samples [16]. Proton (H^1^) nuclear magnetic resonance (NMR) spectrometry was employed to obtain spectral bin values and quantified metabolite levels measured in the same set of stools [12]. Specific differences in the fecal levels of several microbial genera and metabolites were observed between IBS and healthy cohorts. We hypothesized that these microbiota and metabolite data can be subjected to a multivariate discriminant analysis to distinguish between IBS and healthy gut. Multivariate discrimination methods such as partial least squares discriminant analysis (PLS-DA) are very effective at identifying dataset patterns that differentiate samples between different groups. These techniques take into consideration the known group assignment (e.g., IBS-vs-health) for each sample and aim to find a combination of measured variables (e.g., specific microbial abundances or metabolite levels) that can cumulatively separate all or most samples from one group from all or most samples from another group. Indeed, our previous studies revealed that independent microbial and metabolite profiling of fresh stool samples collected from IBS-D and healthy pre-adolescent and adolescent children can separate these samples in the PLS ordination space with good statistical significance (*p* < 0.02) [12, 15]. Techniques such as PLS-DA also offer an intriguing opportunity to classify unknown samples based on the previously constructed model of sample group separation (e.g., IBS-vs-health). This can be used to supplement disease diagnosis in clinical practice. We have thus conducted PLS-DA analyses of fecal microbiota and metabolite datasets obtained previously for the IBS-D (denoted kIBS) and healthy (denoted kHLT) child cohorts [6, 12, 15]. The overall procedure is depicted in Fig. 1. Full microbial and metabolite datasets used to construct PLS-DA models are provided in Additional file 1. Detailed description of methods and statistical procedures is available in Additional file 2.

**Fig. 2:**
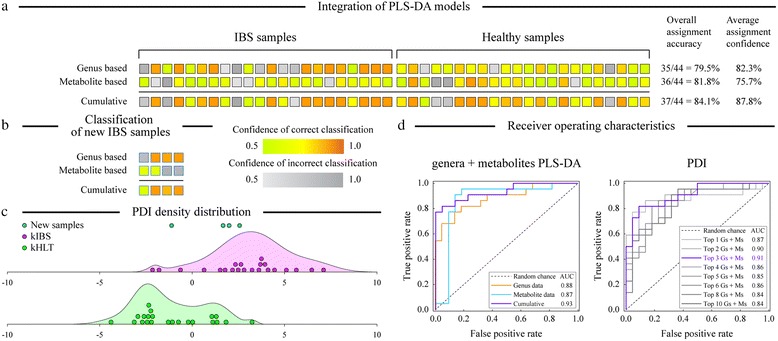
Improvement of sample classification based on the integration of microbiota- and metabolite-based PLS-DA models. **a** Sample classifications are shown as provided by the microbial genus abundance-based PLS-DA model (*top row*), metabolite-based PLS-DA model (*middle row*), and combined Bayesian model (*bottom row*). *Each column* represents a unique sample from IBS and healthy sets as shown. *Each square* is colored according to the group assignment confidence based on the gradient as shown in the legend. Average assignment accuracy and confidence for each model are indicated at the *right* of the figure. **b** Application of the Bayesian integration model to a set of four new IBS-D samples. **c** Density distribution plots of PDI values for IBS-D and healthy adolescent samples. Top three discriminating genera and metabolites were used to compute PDI values. The *X axis* shows the range of PDI values; the *Y axis* represents the density (frequency) of PDI values at each position along the *X* axis. PDI values for individual kIBS and kHLT samples are shown on the plots as *discrete points. Blue points* represent new IBS-D samples. **d** Receiver operating characteristic analysis of PLS-DA models (*left panel*) and patient discrimination indices (*right panel*). *AUC* area under the curve (represents the discrimination ability of each model; higher value equals better discrimination), *G* genus, *M* metabolite

We first generated individual sample classification models based separately on the microbiota and metabolite profiles of the examined samples (Additional file 3 contains class assignment probabilities for each sample). Our PLS-DA model based on the microbial genus abundances in kIBS and kHLT samples achieved 79.5 % accuracy of correct sample classification (sensitivity—72.7 %, specificity—86.4 %, predictive value for IBS (PV_IBS_) = 84.2 %) [15]. The metabolite-based PLS-DA model for the same set of samples attained 81.8 % accuracy of sample group assignment (sensitivity—77.3 %, specificity—86.4 %, PV_IBS_ = 85.0 %). While each individual classification model displayed respectable performance parameters, we hypothesized that combining multiple sample classifications into a joint classifier/predictor can improve prediction accuracy and model robustness. To that goal, we employed an integrative Bayesian approach to combine separate PLS-DA models (one based on metabolite measurements and another based on genus abundance values) into a single classifier as shown in Fig. 1. Combining two models significantly improved our group assignment accuracy and confidence (Fig. 2a): the resulting integrative model achieved an 84.1 % accuracy level with an average 87.8 % confidence of correct sample classification (sensitivity—81.8 %, specificity—86.4 %, PV_IBS_ = 85.7 %). The diagnostic accuracy of the integrative PLS-DA model compared favorably to other IBS diagnostic tools and biomarkers [17]. The combination of the cumulative model’s high positive likelihood ratio (6.02; describes the likelihood of an individual having the disease if the diagnostic test is positive) and low negative likelihood ratio (0.21; describes the likelihood of an individual having the disease if the test is negative) would rank the cumulative genus-metabolite PLS-DA model in the top 3 individual diagnostic tests for IBS [17]. Similar improvement in sample classification was also observed for the combined model based on PLS-DA analyses of the full NMR spectral bin data and microbial phylotype values (see Additional file 4).

To further assess Bayesian classification model performance, the model was applied to the microarray and NMR datasets obtained from fecal samples of four newly recruited IBS-D adolescent patients. Even though individual PLS-DA models were unable to grade all four samples as IBS, the combined microbiota-metabolite PLS-DA model classified fecal samples correctly as IBS type for all new participants (Fig. 2b). The receiver operating characteristic (ROC) analysis presented in Fig. 2d was used to assess the expected performance of PLS-DA models as a clinical diagnostic test. Area under the ROC curve (AUC) values were 0.87, 0.88, and 0.93 for metabolite-, genus-, and integrated metabolite-genus-based PLS-DA classification, respectively, indicating that fecal metabolite-genus PLS-DA classifiers can be expected to perform very well as diagnostic tools. Similar performance characteristics were evident from the ROC analysis of spectral binned-microbial phylotype dataset (see Additional file 4).

To facilitate the application of fecal microbiota- and metabolite-based sample classification in the clinical setting, we also calculated an IBS-vs-health patient discrimination index (PDI) following a recently described strategy [18]. To compute the PDI, we first identified the top discriminating genera and equal number of discriminating metabolites based on the ranks of their PLS weights. We then compared the values of each discriminating variable in a sample to the median value of that variable among all 44 samples of the training dataset. The sum of log_2_ of the ratio between a variable value and its median for discriminating variables was taken to produce the PDI. The complete calculation formula is provided in Additional file 2. The index was designed so that a PDI above zero would indicate that the unknown sample is more likely to be from an IBS-D patient, whereas a PDI value below zero would correspond to samples from healthy individuals. Figure 2d presents the ROC analysis of expected diagnostic performance of different PDIs based on the number of top discriminating variables used. The ROC analysis indicated that inclusion of the top three genera (*Parasporobacterium*, *Oxalobacter*, and *Enterobacter*) and top three metabolites (formate, pyruvate, and glucose) in PDI presented the best discriminating power (highest AUC value) compared to other choices. Figure 2c shows the density distributions of the top three “genera + metabolites” PDI values for the kIBS and kHLT samples. While the discriminatory power of PDI is lower than that of the Bayesian classification model described above, the median PDI was nevertheless significantly different between healthy and IBS groups (3.0 and −1.7 median PDI for kIBS and kHLT groups, respectively; *p* < 0.001 based on Mood’s median test of significance). We also calculated PDI values for new IBS-D samples. Three of the four samples had positive PDI values (2.6, 2.0, and 1.8; PDI > 0 indicates higher likelihood of IBS diagnosis) and one sample had a negative PDI (−1.1, see Fig. 2c). These results indicate that PDI can be used to facilitate classification of patients with IBS-D.

Because the diagnosis of IBS still presents several challenges [14], additional non-subjective diagnostic tools can significantly facilitate clinical assessment of a patient. The combined metabolite- and microbiota-based IBS-vs-health classification model described above does not rely on the identification of a single unique biomarker of the disease; rather, it assembles a set of recognized fecal microbial and metabolite differences that are used jointly to confidently distinguish between IBS-D and health. The values of these microbial taxons and metabolites may or may not correlate to one another among samples, but all provide good discrimination between two analyzed cohorts. This approach of relying on a set of variables makes the model robust and able to classify correctly even some outlier samples. Additionally, the model can reveal the individual variables (microbial taxa and metabolites) that contribute most to the IBS-vs-health discrimination; presumably, these are important in the etiology of the disease. In our models, the top discriminating genera included *Parasporobacterium*, *Oxalobacter*, and *Enterobacter*; the top discriminating metabolites were formate, pyruvate, glucose, lysine, and tyrosine (see Additional file 5). Previously conducted statistical tests also indicated that the levels of these genera and metabolites were significantly different between fecal samples from healthy children and children with IBS [6, 12]. While very little information is currently available for the discriminating microbial genera, the higher levels of several amino acids including lysine and tyrosine point to an increased proteolysis in IBS-D. At the same time, increased levels of carbohydrate degradation intermediates such as glucose in the stools of IBS-D children are likely indicators of the incomplete fermentation process in the gut of these subjects [12]. This finding is consistent with our previously revealed loss of microbe-microbe and microbe-metabolite associations in this cohort of IBS children [12, 15].

While the patient’s symptom evaluation will undoubtedly remain a critical part of IBS diagnosis, the ability to utilize quantifiable measurements of the components within the gut environment should facilitate the distinction between the healthy and IBS gut. Knowledge of specific discriminatory microbes and metabolites in the patient gut can also assist in the choice of the most appropriate therapy, for example, the selection of antimicrobial therapy, dietary management, pre- and probiotic treatments, or the design of personalized symbiotic mixtures in the future. While further analyses are needed to build a generalized fecal diagnostic model to distinguish different subtypes of IBS from Crohn’s disease, ulcerative colitis, other gastrointestinal disorders, and health, we are optimistic that simultaneous fecal microbiota and metabolite profiling, a non-intrusive, quantitative approach, may prove useful in enhancing the management of IBS in clinical practice.

## Availability of supporting data

The datasets of relative microbial abundances and metabolite levels (abundances of 115 genera and 19 metabolites) measured in the set of 44 fecal samples were available from our previous studies [6, 12] and are provided in Additional file 1. All experimentally available data were used in PLS-DA modeling. The datasets of microbial phylotypes and NMR spectral bins were taken from the same sources. The datasets supporting the results of this article are available in Additional files 2, 3, 4, and 5.

## Additional files

Additional file 1:
**Complete PLS-DA input datasets.** This file contains input datasets that were used to generate PLS-DA models. (XLSX 591 kb) Additional file 2:
**Detailed methods and statistical procedures.** (PDF 126 kb) Additional file 3:
**Class assignment probabilities for each sample obtained in PLS-DA models.** This table shows the class assignment probabilities for each sample obtained in PLS-DA models. (PDF 247 kb) Additional file 4:
**Improvement of sample classification based on the integration of microbial phylotype and NMR spectral bin-based PLS-DA models.** (**a**). Sample classifications are shown as provided by the microbial phylotype abundance-based PLS-DA model (top row), NMR spectral bin-based PLS-DA model (middle row), and combined Bayesian model (bottom row). (**b**). Application of the phylotype + spectral bin Bayesian integration model to a set of four new IBS-D samples. (**c**). Receiver operating characteristic analysis of the phylotype + spectral bin PLS-DA models. (TIF 767 kb) Additional file 5:
**Assigned weights for all variables applied to generate PLS discrimination between IBS-D and healthy groups.** (PDF 38 kb)

## Abbreviations

IBS-D: diarrhea-predominant IBS
kHLT: cohort of healthy children
kIBS: cohort of IBS children
NMR: nuclear magnetic resonance
PDI: patient discrimination index
PLS-DA: partial least squares discriminant analysis
PV: predictive value
ROC: receiver operating characteristics
