# Supplementary material for: Simultaneous fecal microbial and metabolite profiling enables accurate classification of pediatric irritable bowel syndrome
Source: Microbiome. 2015 Dec 9;3:73. doi: 10.1186/s40168-015-0139-9 (PMC4675077; doi:10.1186/s40168-015-0139-9)
Supplement: Additional file 2: — Detailed methods and statistical procedures. (PDF 126 kb) [file 40168_2015_139_MOESM2_ESM.pdf]

Simultaneous fecal microbial and metabolite profiling enables accurate classification of pediatric irritable bowel syndrome

Vijay Shankar, Nicholas V. Reo, and Oleg Paliy

METHODS AND STATISTICAL PROCEDURES

Study cohort. The fecal samples were obtained from 22 healthy pre- and adolescent volunteers (designated kHLT for healthy kids; n=22; age range: 11-18 years, average: 12.6 years; 10 males, 12 females) and from 22 children diagnosed with diarrhea-predominant IBS (designated kIBS; n=22; age range: 8-18 years, average: 13.2 years; 10 males, 12 females). To validate the constructed IBS-vs-health classification model, four additional children diagnosed with diarrhea-predominant IBS were recruited into the study. The study was approved by the Wright State University and Dayton Children's Hospital IRB committees. Diagnosis of the IBS, inclusion and exclusion criteria, and the stool collection procedure were described previously [1]. Phylogenetic Microbiota Array was used to obtain quantitative microbial phylotype and genus abundance values [2]. Proton (H^1) NMR was employed to obtain spectral bin values and quantified metabolite levels measured in the same set of fecal samples [3].

Statistical data analyses. To build an integrative model of sample classification based on both the metabolite and microbiota abundance data, we first ran separate PLS discriminant analyses to obtain posterior probabilities of sample classification to each group (kHLT and kIBS) based on each type of data. Because PLS can use predictor variables that are collinear and not independent [4], it was deemed a good choice of discriminatory algorithm to be applied to our datasets. The fractional microbial and metabolic datasets provided in Additional file 1 were processed through a chord transformation [5] and were then subjected to PLS algorithm. Two different integrative Bayesian PLS-DA models were developed and tested – one based on the specific measured metabolites and microbial genera, and another based on the complete NMR spectral profile combined with complete phylotype profile of each sample. To provide PLS-DA based classification for each sample, a K-fold cross-validation with K=22 was used [6]. All samples were randomly divided into 22 sets, iteratively 1 set of samples was removed, and *de novo* PLS-DA classification model was generated based on the data for the remaining samples. The omitted samples were then classified based on that PLS-DA model; thus, classification of each sample was based on the model developed without that sample participation [6]. Overall accuracy of PLS-DA modeling was then calculated as the number of correctly classified samples among all 44. Twenty iterations of K-fold validation algorithm were run, and the classification accuracy and confidence numbers were averaged across all iterations. Note that the choice of K number of folds influences the obtained estimates of accuracy and confidence. As the K decreases, higher fractions of overall sample set are removed from the training subset, which results in higher estimation bias and variance [7].

To classify each sample, PLS-DAs generated likelihood values that a sample i (s_i) belonged to class j (G_j, either IBS or healthy) in dataset h (D_h, either microbiota (M) or metabolite (C)). Each sample was then assigned to the class with the larger posterior probability that was calculated as

$$P(s_i,\, D_h \mid G_j)P(G_j) \Big/ \sum_j P(s_i,\, D_h \mid G_j)P(G_j), \quad h \in [C, M]$$

where $P(G_j)$ is the prior probability of class j (in our dataset, $P(G_{IBS})=P(G_{HLT})=0.5$; in the overall population with 20% IBS prevalence rate prior probabilities are $P(G_{IBS})=0.2$ and $P(G_{HLT})=0.8$). Performance of PLS modeling was described through calculations of model accuracy, sensitivity (defined as percent correct classification of kIBS samples), specificity (defined as percent correct classification of kHLT samples), positive and negative likelihood ratios (defined as [sensitivity]/[1-specificity] and [1-sensitivity]/[specificity], respectively), as well as its power to predict IBS (defined as the ratio of [true kIBS positives]/[true kIBS positives+false kIBS positives]). Posterior probabilities of sample classification for each PLS-DA model are provided in Supplementary Table S2.

A Bayesian integration approach was then utilized to incorporate both PLS-DA classifications into one cumulative model following a recently described method by Webb-Robertson and colleagues [8]. Specifically, the integrative group membership likelihoods were defined as products of individual likelihoods: $P(s_i, D_{MC} \mid G_j) = P(s_i, D_M \mid G_j) * P(s_i, D_C \mid G_j)$, where D_{MC} is a combined microbiota and metabolite dataset [8]. The posterior class probabilities in the integrative model were calculated as

$$P(s_i, D_{MC} \mid G_j)P(G_j) \Big/ \sum_j P(s_i, D_{MC} \mid G_j)P(G_j)$$

Sample class assignment was then defined as $s_i \in G_{CUM}$, where $G_{CUM} = \max[P(G_j \mid s_i, D_{MC})]$.

Patient discrimination index (PDI) was developed as a more straightforward IBS-vs-health differentiating measure better suited for clinical application. Based on the ROC analysis (see Figure 2D), the top three discriminating genera and top three discriminating metabolites (defined as three variables with the largest weights in each PLS-DA model) were used to define PDI as:

$$PDI(s_i) = \sum_{\substack{k \in IBS \\ l \in IBS}} \left(log_2\left(\frac{m_{ik}+1}{\widetilde{m_k}+1}\right) + log_2\left(\frac{c_{il}+1}{\widetilde{c_l}+1}\right) \right) - \sum_{\substack{k \in HLT \\ l \in HLT}} \left(log_2\left(\frac{m_{ik}+1}{\widetilde{m_k}+1}\right) + log_2\left(\frac{c_{il}+1}{\widetilde{c_l}+1}\right) \right)$$

where s_i is sample i, m_{ik} and c_{il} are the abundances of discriminating genus k and metabolite l in sample i, *IBS* and *HLT* are subsets of discriminating fecal genera and metabolites that were enriched in IBS-D and health, respectively, and $\widetilde{m_k}$ and $\widetilde{c_l}$ are the medians of the genus k and metabolite l abundances, respectively, taken from the training dataset. PDI values were calculated for each of the original samples and were compiled into a PDI density distribution for kIBS and kHLT sets.

References

1. Rigsbee L, Agans R, Shankar V, Kenche H, Khamis HJ, Michail S, Paliy O: **Quantitative profiling of gut microbiota of children with diarrhea-predominant irritable bowel syndrome.** *Am J Gastroenterol* 2012, **107:**1740–1751.
2. Rigsbee L, Agans R, Foy BD, Paliy O: **Optimizing the analysis of human intestinal microbiota with phylogenetic microarray.** *FEMS Microbiol Ecol* 2011, **75:**332-342.
3. Shankar V, Homer D, Rigsbee L, Khamis HJ, Michail S, Raymer M, Reo NV, Paliy O: **The networks of human gut microbe-metabolite associations are different between health and irritable bowel syndrome.** *The ISME journal* 2015, **9:**1899-1903.
4. Tobias RD: **An introduction to partial least squares regression.** In *Proc Ann SAS Users Group Int Conf, 20th, Orlando, FL*. Citeseer; 1995: 2-5.
5. Legendre P, Gallagher ED: **Ecologically meaningful transformations for ordination of species data.** *Oecologia* 2001, **129:**271-280.
6. Simon RM, Subramanian J, Li MC, Menezes S: **Using cross-validation to evaluate predictive accuracy of survival risk classifiers based on high-dimensional data.** *Briefings in bioinformatics* 2011, **12:**203-214.
7. Burman P: **A comparative study of ordinary cross-validation, nu-fold cross-validation and the repeated learning-testing methods.** *Biometrika* 1989, **76:**503-514.
8. Webb-Robertson BJ, McCue LA, Beagley N, McDermott JE, Wunschel DS, Varnum SM, Hu JZ, Isern NG, Buchko GW, McAteer K, et al: **A bayesian integration model of high-throughput proteomics and metabolomics data for improved early detection of microbial infections.** *Pacific Symposium on Biocomputing* 2009**:**451-463.
