# Supplementary material for: Simultaneous fecal microbial and metabolite profiling enables accurate classification of pediatric irritable bowel syndrome
Source: Microbiome. 2015 Dec 9;3:73. doi: 10.1186/s40168-015-0139-9 (PMC4675077; doi:10.1186/s40168-015-0139-9)
Supplement: Additional file 3: — Class assignment probabilities for each sample obtained in PLS-DA models. This table shows the class assignment probabilities for each sample obtained in PLS-DA models. (PDF 247 kb) [file 40168_2015_139_MOESM3_ESM.pdf]

This table shows the class assignment probabilities for each sample obtained in PLS-DA models

Sample	Microbial genera		NMR metabolites		Microbial phylotypes		NMR bins	
	kIBS prob	kHLT prob	kIBS prob	kHLT prob	kIBS prob	kHLT prob	kIBS prob	kHLT prob
kIBS01	0.17	0.83	0.68	0.32	0.66	0.34	0.99	0.01
kIBS02	0.99	0.01	0.48	0.52	0.56	0.44	0.62	0.38
kIBS03	0.66	0.34	0.10	0.90	0.91	0.09	0.39	0.61
kIBS04	1.00	0.00	0.85	0.15	0.99	0.01	0.17	0.83
kIBS05	0.69	0.31	0.59	0.41	0.50	0.50	0.60	0.40
kIBS06	0.95	0.05	0.60	0.40	0.48	0.52	0.62	0.38
kIBS07	1.00	0.00	0.75	0.25	0.98	0.02	0.57	0.43
kIBS08	0.49	0.51	0.76	0.24	0.47	0.53	0.95	0.05
kIBS09	0.12	0.88	0.47	0.53	0.54	0.46	0.61	0.39
kIBS10	0.73	0.27	0.46	0.54	0.34	0.66	0.63	0.37
kIBS11	0.28	0.72	0.84	0.16	0.47	0.53	0.67	0.33
kIBS12	0.98	0.02	0.71	0.29	0.72	0.28	0.59	0.41
kIBS13	0.38	0.62	0.88	0.12	0.65	0.35	0.96	0.04
kIBS14	0.26	0.74	0.70	0.30	0.25	0.75	0.76	0.24
kIBS15	0.99	0.01	0.80	0.20	0.78	0.22	0.45	0.55
kIBS16	0.96	0.04	0.70	0.30	0.76	0.24	0.63	0.37
kIBS17	1.00	0.00	0.41	0.59	0.99	0.01	0.55	0.45
kIBS18	0.97	0.03	0.88	0.12	0.97	0.03	0.96	0.04
kIBS19	0.55	0.45	0.78	0.22	0.51	0.49	0.59	0.41
kIBS20	0.99	0.01	0.81	0.19	0.70	0.30	0.98	0.02
kIBS21	0.99	0.01	0.84	0.16	0.75	0.25	0.55	0.45
kIBS22	0.99	0.01	0.69	0.31	0.94	0.06	0.80	0.20
kHLT01	0.19	0.81	0.35	0.65	0.28	0.72	0.25	0.75
kHLT02	0.06	0.94	0.01	0.99	0.04	0.96	0.05	0.95
kHLT03	0.57	0.43	0.28	0.72	0.27	0.73	0.20	0.80
kHLT04	0.22	0.78	0.88	0.12	0.37	0.63	0.15	0.85
kHLT05	0.25	0.75	0.88	0.12	0.84	0.16	0.84	0.16
kHLT06	0.07	0.93	0.12	0.88	0.03	0.97	0.06	0.94
kHLT07	0.18	0.82	0.00	1.00	0.31	0.69	0.28	0.72
kHLT08	0.11	0.89	0.06	0.94	0.24	0.76	0.19	0.81
kHLT09	0.58	0.42	0.13	0.87	0.19	0.81	0.09	0.91
kHLT10	0.18	0.82	0.18	0.82	0.15	0.85	0.13	0.87
kHLT11	0.20	0.80	0.11	0.89	0.46	0.54	0.43	0.57
kHLT12	0.30	0.70	0.27	0.73	0.43	0.57	0.65	0.35
kHLT13	0.07	0.93	0.35	0.65	0.26	0.74	0.15	0.85
kHLT14	0.39	0.61	0.36	0.64	0.51	0.49	0.31	0.69
kHLT15	0.11	0.89	0.06	0.94	0.24	0.76	0.16	0.84
kHLT16	0.16	0.84	0.55	0.45	0.10	0.90	0.68	0.32
kHLT17	0.30	0.70	0.34	0.66	0.45	0.55	0.25	0.75
kHLT18	0.08	0.92	0.21	0.79	0.04	0.96	0.15	0.85
kHLT19	0.84	0.16	0.33	0.67	0.91	0.09	0.95	0.05
kHLT20	0.07	0.93	0.19	0.81	0.13	0.87	0.19	0.81
kHLT21	0.27	0.73	0.41	0.59	0.08	0.92	0.26	0.74
kHLT22	0.29	0.71	0.19	0.81	0.17	0.83	0.20	0.80
