# Supplementary figures and images for: Simultaneous fecal microbial and metabolite profiling enables accurate classification of pediatric irritable bowel syndrome
Source: Microbiome. 2015 Dec 9;3:73. doi: 10.1186/s40168-015-0139-9 (PMC4675077; doi:10.1186/s40168-015-0139-9)

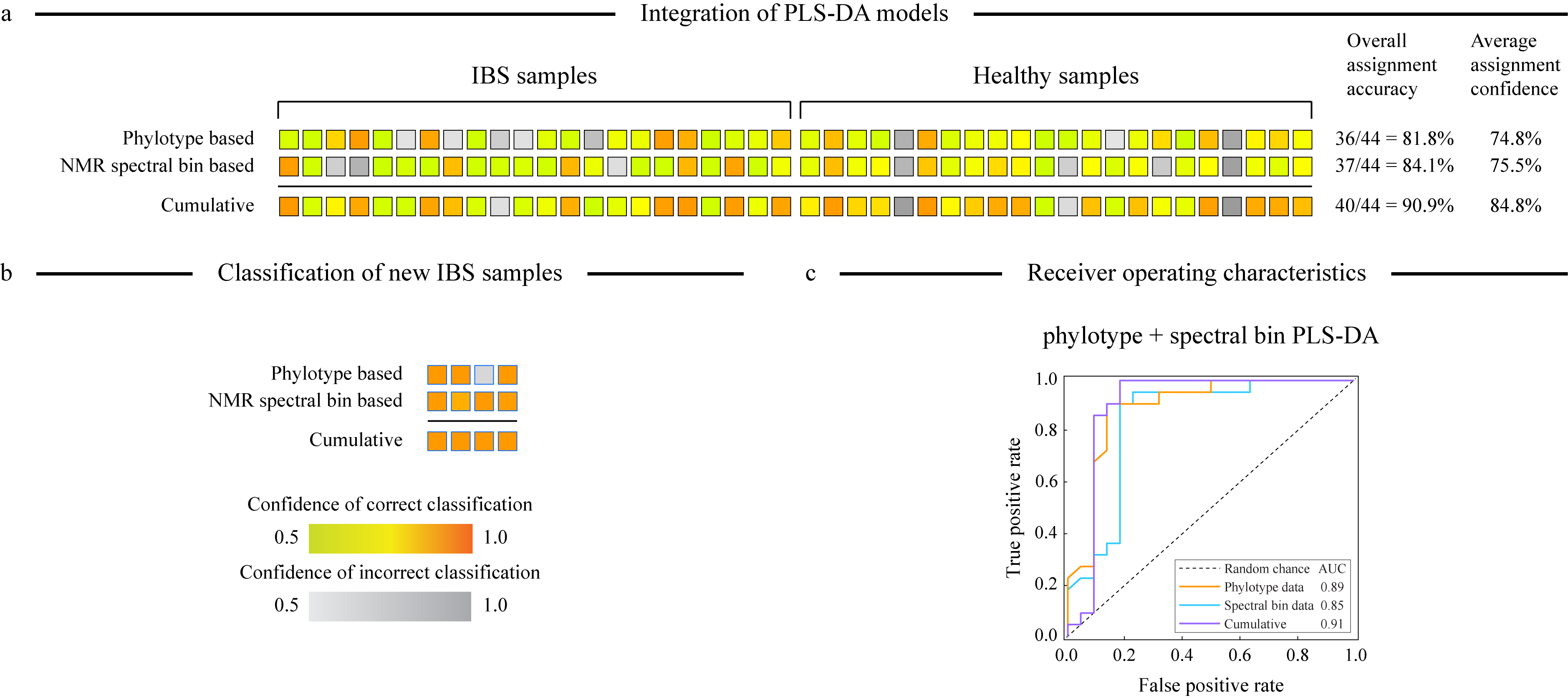

Supplement: Additional file 4: — Improvement of sample classification based on the integration of microbial phylotype and NMR spectral bin-based PLS-DA models. (a). Sample classifications are shown as provided by the microbial phylotype abundance-based PLS-DA model (top row), NMR spectral bin-based PLS-DA model (middle row), and combined Bayesian model (bottom row). (b). Application of the phylotype + spectral bin Bayesian integration model to a set of four new IBS-D samples. (c). Receiver operating characteristic analysis of the phylotype + spectral bin PLS-DA models. (TIF 767 kb) [file 40168_2015_139_MOESM4_ESM.tif]
