# Supplementary material for: Simultaneous fecal microbial and metabolite profiling enables accurate classification of pediatric irritable bowel syndrome
Source: Microbiome. 2015 Dec 9;3:73. doi: 10.1186/s40168-015-0139-9 (PMC4675077; doi:10.1186/s40168-015-0139-9)
Supplement: Additional file 5: — Assigned weights for all variables applied to generate PLS discrimination between IBS-D and healthy groups. (PDF 38 kb) [file 40168_2015_139_MOESM5_ESM.pdf]

Genera	Weights	|Weights|
Parasporobacterium	0.300	0.300
Oxalobacter	0.219	0.219
Enterobacter	0.200	0.200
Garciella	-0.193	0.193
Gemella	0.191	0.191
Staphylococcus	0.188	0.188
Alistipes	-0.186	0.186
Dorea	0.182	0.182
Fusibacter	-0.179	0.179
Solobacterium	0.175	0.175
Prevotella	0.171	0.171
Bradyrhizobium	0.170	0.170
Anaerotruncus	-0.169	0.169
Dehalobacter	-0.166	0.166
Acidaminobacter	-0.163	0.163
Caminicella	-0.151	0.151
Acetivibrio	-0.147	0.147
Bifidobacterium	-0.146	0.146
Veillonella	0.146	0.146
Lactococcus	-0.146	0.146
Brachybacterium	0.143	0.143
Anaerofilum	-0.143	0.143
Klebsiella	0.141	0.141
Anaerofustis	0.140	0.140
Megasphaera	0.138	0.138
Anaerosinus	-0.137	0.137
Anaerovorax	-0.135	0.135
Desulfovibrio	-0.133	0.133
Mitsuokella	0.130	0.130
Peptococcus	-0.130	0.130
Acidaminococcus	0.129	0.129
Syntrophococcus	0.126	0.126
Oxobacter	-0.126	0.126
Geopsychrobacter	-0.124	0.124
Turicibacter	-0.123	0.123
Tannerella	-0.123	0.123
Raoultella	0.118	0.118
Actinomyces	0.118	0.118
Succiniclasticum	-0.118	0.118
Lactobacillus	0.118	0.118
Anaerococcus	-0.118	0.118
Escherichia	0.116	0.116
Pelotomaculum	-0.114	0.114
Mogibacterium	0.114	0.114
Acetanaerobacterium	-0.114	0.114
Ruminococcus	0.114	0.114
Succinivibrio	0.112	0.112
Verrucomicrobium	-0.111	0.111
Allisonella	0.108	0.108
Lachnobacterium	-0.107	0.107
Mahella	0.106	0.106

Metabolites	Weights	|Weights|
Formate	0.555	0.555
Pyruvate	-0.441	0.441
Glucose	0.408	0.408
Lysine	0.402	0.402
Tyrosine	0.401	0.401
Methylamine	0.366	0.366
Leucine	0.304	0.304
Lactate	0.255	0.255
Valine	0.241	0.241
Fumarate	0.227	0.227
Alanine	0.213	0.213
Acetate	-0.207	0.207
Succinate	0.133	0.133
Butyrate	0.073	0.073
Proprionate	-0.042	0.042
Valerate	-0.024	0.024
Dimethylamine	0.021	0.021
Cholate-BA	0.014	0.014
Isoleucine	0.014	0.014

Only variables with non-zero weights are shown

Genera	Weights	|Weights|
Catenibacterium	0.105	0.105
Terasakiella	-0.103	0.103
Rothia	0.103	0.103
Shigella	0.100	0.100
Roseburia	0.100	0.100
Peptostreptococcus	0.097	0.097
Brachyspira	-0.097	0.097
Ralstonia	0.096	0.096
Kerstersia	0.096	0.096
Rhodanobacter	0.096	0.096
Bulleidia	0.096	0.096
Fusobacterium	0.096	0.096
Paucimonas	0.093	0.093
Clostridium	-0.091	0.091
Bryantella	0.089	0.089
Collinsella	0.086	0.086
Coprobacillus	0.085	0.085
Roseospirillum	-0.083	0.083
Lachnospira	-0.082	0.082
Butyrivibrio	0.081	0.081
Leptospira	0.078	0.078
Yersinia	0.076	0.076
Streptococcus	0.076	0.076
Phascolarctobacterium	0.074	0.074
Parvimonas	0.072	0.072
Campylobacter	0.064	0.064
Anaerostipes	0.064	0.064
Corynebacterium	0.058	0.058
Holdemania	-0.056	0.056
Papillibacter	0.055	0.055
Caloranaerobacter	-0.055	0.055
Rikenella	-0.052	0.052
Dialister	0.048	0.048
Enterococcus	-0.048	0.048
Eubacterium	0.044	0.044
Limnobacter	-0.037	0.037
Oribacterium	0.034	0.034
Slackia	-0.034	0.034
Anaerophaga	-0.034	0.034
Faecalibacterium	0.032	0.032
Herbaspirillum	-0.032	0.032
Sporobacter	-0.029	0.029
Coprococcus	-0.023	0.023
Acinetobacter	-0.019	0.019
Mesorhizobium	-0.019	0.019
Bacteroides	-0.018	0.018
Victivallis	-0.017	0.017
Subdoligranulum	-0.015	0.015
Haemophilus	0.015	0.015
Eggerthella	-0.014	0.014
Sutterella	0.002	0.002
